# Femoral neck-specific cortical thickness predicts internal fixation complications across adults with implications for elderly surgical decision-making

**DOI:** 10.3389/fmed.2026.1927915

**Published:** 2026-08-27

**Authors:** Zhiyuan Fan, Bohao Yin, Chenjun Liu, Hui Sun, Wei Zhang

**Affiliations:** Department of Orthopaedic Surgery, Shanghai Sixth People’s Hospital Affiliated to Shanghai Jiao Tong University School of Medicine, Shanghai, China

**Keywords:** aged, cortical bone, decision support techniques, femoral neck fractures, internal fixation, postoperative complications, risk assessment, tomography

## Abstract

**Background:**

Chronological age is an unreliable sole criterion for selecting internal fixation (IF) versus arthroplasty in femoral neck fractures (FNFs). This study evaluated whether the femoral neck cortical thickness index (FN-CTI), a CT-based measure of local bone quality, predicts complications after internal fixation in patients spanning a wide age range. We placed particular emphasis on the utility of FN-CTI for decision-making in elderly patients and also assessed whether a composite risk score could further enhance clinical decision-making.

**Methods:**

We retrospectively analyzed 219 FNF patients treated with IF (minimum 24-month follow-up). Independent predictors were identified via multivariable Cox regression. Receiver operating characteristic (ROC) curves, Net Reclassification Improvement (NRI), and Decision Curve Analysis (DCA) were used to evaluate performance and clinical utility. A point-based risk score was constructed from regression coefficients.

**Results:**

Complications occurred in 47 patients (21.46%). FN-CTI (per 0.1 increase: HR = 0.059, *p* < 0.001), modified Pauwels angle (HR = 1.035, *p* = 0.026), and BMI (HR = 1.106, *p* = 0.023) were independent predictors. FN-CTI achieved an AUC of 0.723 (cutoff: 0.12). The composite risk score (0--5 points) stratified patients into low- and high-risk groups with log-rank *p* < 0.001. DCA confirmed superior net benefit across clinically relevant threshold probabilities.

**Conclusion:**

In this derivation cohort, lower FN-CTI was independently associated with a higher rate of mechanical complications after internal fixation in adults with femoral neck fractures. The same direction of effect was observed in the small elderly subgroup. The composite score improved risk stratification compared with individual predictors alone. However, whether high-risk patients would benefit from primary arthroplasty remains unknown and requires future comparative studies. Prospective external validation is required before clinical adoption, particularly in unselected older populations.

**Trial registration:**

This retrospective cohort study was approved by the institutional review board of our center and registered at www.chictr.org.cn (ChiCTR1900023156).

## 1 Introduction

Although the global incidence of hip fractures varies by region, it continues to rise in many countries worldwide (1). Anatomically, femoral neck fractures (FNFs) account for approximately 3.6% of all systemic fractures and constitute half of all hip fractures, with substantial associated morbidity and disability (2). Surgical options for FNFs include internal fixation (e.g., multiple cancellous screws or sliding hip screw) and arthroplasty (hemiarthroplasty or total hip arthroplasty) (3, 4). Internal fixation remains the primary treatment modality for young and active patients. However, an increasing number of older patients are also considered for internal fixation when their bone quality is preserved, underscoring the need for a reliable biological marker to guide this decision across the adult age spectrum.

Currently, advanced age (typically ≥65 years) is frequently used as a relative contraindication for internal fixation (3, 5). In patients younger than 65 years, internal fixation is generally preferred; in those aged 65 years or older, arthroplasty is often recommended. However, this binary age-based paradigm is increasingly recognized as overly simplistic. Some younger patients experience fixation failure, whereas many older patients with good bone quality achieve successful healing with internal fixation. The complication rate after internal fixation of FNFs remains alarmingly high—including avascular necrosis of the femoral head (10–23%), nonunion (8–19%), malunion (7.1%), and implant failure (9.7%)—substantially exceeding that of other fractures (6, 7). Numerous factors influence prognosis, including displacement severity (Garden classification), fracture obliquity (modified Pauwels angle), and the patient’s physiological bone condition (1). Therefore, it is unreasonable to rely solely on chronological age for surgical decision-making, and its correlation with complications warrants rigorous re-evaluation.

The proximal femoral cortical thickness index (PF-CTI) has been validated as a radiographic predictor of bone mineral density and fracture risk (8–12). However, PF-CTI measures cortical thickness at the femoral diaphysis, 10 cm distal to the lesser trochanter, which poorly reflects the local cortical status at the femoral neck—the actual fracture site. Currently, few radiological parameters are specifically designed to predict post-fixation complications at the femoral neck. To address this gap, we propose the femoral neck cortical thickness index (FN-CTI), defined as the ratio of cortical thickness to total neck width measured perpendicular to the femoral neck axis. Unlike PF-CTI, FN-CTI directly quantifies the cortical bone stock at the fracture site, potentially offering superior predictive value for fixation outcomes. We also explored two parameters related to the principal compressive trabeculae (PCT-A and PCT-R) based on the distribution characteristics of femoral neck trabecular architecture (13, 14). However, preliminary analysis demonstrated no evidence of association with these trabecular parameters; consequently, our investigation focused on the cortical index.

The primary aims of this study were: (1) to validate FN-CTI as an independent predictor of complications after internal fixation of FNFs; (2) to compare its predictive performance with that of PF-CTI, BMI, and modified Pauwels angle; and (3) to develop a simple, clinically actionable risk stratification scoring system integrating these independent predictors to estimate complication risk after internal fixation across adults, and provides preliminary insights that may be relevant for geriatric surgical decision-making, though this requires confirmation in larger elderly-specific cohorts.

## 2 Materials and methods

### 2.1 Study design and ethics

This retrospective cohort study was approved by the institutional review board of Shanghai Sixth People’s Hospital (Approval No. 2023-032-KY). The requirement for individual written informed consent was formally waived by the ethics committee due to the retrospective nature of the study and the use of anonymized, de-identified patient data. The study complied with the Code of Ethics of the World Medical Association (Declaration of Helsinki) for experiments involving humans.

This study was registered at www.chictr.org.cn (ChiCTR1900023156) on January 15, 2019, prior to patient enrollment (first patient enrolled: February 1, 2020). The study design and analysis plan were prespecified in the registration protocol (version 1.0, dated January 10, 2019). No major deviations from the registered protocol occurred. The full protocol is available at: https://www.chictr.org.cn.

### 2.2 Participants

**Figure 1:**
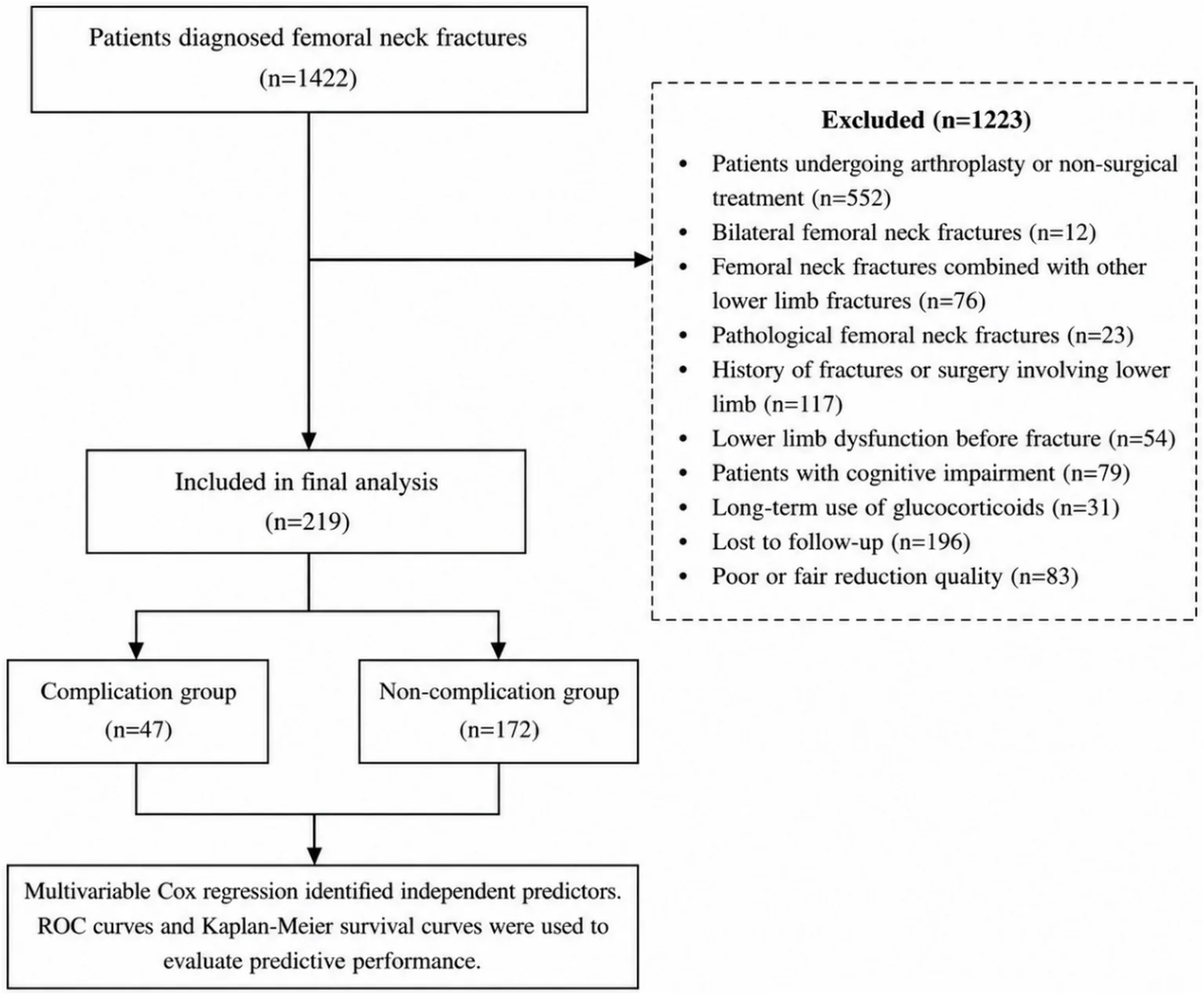
Study flow chart.

Patients diagnosed with femoral neck fractures at our hospital (level I trauma center) between January 2020 and September 2021 were retrospectively screened (Figure 1). Inclusion criteria: patients older than 18 years; patients with femoral neck fractures undergoing internal fixation. Exclusion criteria: patients undergoing arthroplasty or non-surgical treatment; poor or fair reduction quality of internal fixation immediate postoperatively; bilateral femoral neck fractures; femoral neck fractures combined with other lower limb fractures; pathological fractures; history of fractures or surgery involving the hip joint or lower limb; lower limb dysfunction before fracture; patients with cognitive impairment; drug or alcohol abuse; primary metabolic bone diseases (hyper- or hypoparathyroidism, renal osteodystrophy, osteogenesis imperfecta), renal failure, or immune diseases; chronic glucocorticoid use (>3 months); loss to follow-up; incomplete clinical and radiological evaluation information during follow-up. Diabetes mellitus and hypertension were not exclusion criteria but were recorded as baseline comorbidities.

All patients underwent internal fixation using either multiple cannulated screws (n = 168, 76.7%) or a sliding hip screw (n = 51, 23.3%). For cannulated screw fixation, a triangular configuration was used in all cases, with 6.5-mm or 7.3-mm screws placed under fluoroscopic guidance. All surgeries were performed by one of five senior trauma surgeons (each with >10 years of experience). Fracture reduction quality was graded postoperatively using Haidukewych criteria; only patients with good reduction were included. Capsulotomy was performed in 18 patients (8.2%) with high-energy injuries to decompress the joint. Postoperative rehabilitation followed a standardized protocol: non-weight-bearing for 6 weeks, followed by progressive partial and full weight-bearing as tolerated. These variables were not entered into the multivariable model due to the limited event count (n = 47), which constrained the number of covariates that could be reliably included.

Of the 1,422 screened patients, 1,223 were excluded: arthroplasty or non-surgical treatment (*n* = 552), loss to follow-up (*n* = 196), history of lower limb fracture/surgery (*n* = 117), poor or fair reduction quality (*n* = 83), cognitive impairment (*n* = 79), lower limb dysfunction before fracture (*n* = 54), long-term glucocorticoid use (*n* = 31), pathological fractures (*n* = 23), bilateral fractures (*n* = 12), and other lower limb fractures (*n* = 76). The final cohort thus represents a selected group with satisfactory initial fixation and complete follow-up, limiting generalizability to unselected geriatric populations.

All patients underwent anteroposterior radiographs of the pelvis including the proximal femur and computed tomography scanning of the hip joints using a standard technique. Patients whose anteroposterior pelvic radiographs did not visualize the proximal femur 10 cm below the lesser trochanter were excluded during screening. The quality of fracture reduction was graded based on the published criteria by Haidukewych et al. (15).

After collection of medical history and follow-up information, the screening processes were blindly performed by two independent orthopedic surgeons. They agreed on an evaluation by consensus if there was disagreement.

The age distribution of this cohort was as follows: 119 patients (54.3%) were under 55 years, 68 (31.1%) were aged 55–64 years, and 32 (14.6%) were aged 65 years or older. While the majority of patients were younger than 65 years, this reflects the clinical reality that internal fixation is predominantly offered to younger and middle-aged patients in our institution; elderly patients with poor bone quality are preferentially treated with arthroplasty. Nevertheless, the 32 elderly patients (≥65 years) in this cohort who underwent internal fixation allowed us to perform a preliminary, exploratory subgroup analysis (Supplementary Table S1). In this subgroup, there were 12 complication events (37.5%). The analysis demonstrated a consistent direction of effect for FN-CTI (per 0.1 increase: HR = 0.071, 95% CI: 0.018–0.285, *p* = 0.002), with covariates including BMI, modified Pauwels angle, and FN-CTI. This exploratory finding suggests that the protective effect of FN-CTI is not limited to younger patients and provides hypothesis-generating evidence for its potential utility in geriatric populations. However, due to the small sample size and limited number of events, these results should be considered preliminary and require dedicated validation in larger elderly cohorts. Dedicated external validation in larger elderly cohorts is ongoing.

### 2.3 Outcome measures

The primary clinical outcome of this study was complication at a minimum of 24 months after fixation. Complications included AVN of the femoral head, fracture nonunion, and internal fixation failure. Time zero was defined as the date of surgery. For time-to-event analysis, the event date was assigned as the date of radiographic confirmation for AVN and fixation failure, and as 6 months post-surgery for nonunion diagnosis. Patients who underwent reoperation or conversion arthroplasty were censored at the date of reoperation only if the procedure was performed for reasons other than the predefined complications (e.g., elective implant removal, infection, or post-traumatic osteoarthritis without preceding nonunion, AVN, or fixation failure); if the reoperation was specifically for nonunion, AVN, or fixation failure, patients were counted as having had the event at the time of radiographic diagnosis, which preceded the reoperation. Patients who died were censored at the date of death and treated as a competing risk in cause-specific analyses. Patients without complications were censored at their last clinical follow-up. This approach ensures that all events were captured at their time of diagnosis rather than at the time of subsequent surgical intervention.

The criteria for each complication were as follows: Fracture nonunion was considered present if the fracture line was grossly visible at 6 months postoperatively (16)^.^ The radiographic criteria of Ficat and Arlet were used to diagnose avascular necrosis of the femoral head (17). Fixation failure was defined as varus collapse (>10°), fracture displacement (>5 mm), or femoral neck shortening (>10 mm) (16, 18). To detect fixation failure, the parameters of the injured hip joint were compared with those of the uninjured side on the latest follow-up anteroposterior radiographs. The difference in neck-shaft angles between the two hip joints was measured to identify varus collapse. Femoral neck shortening was assessed according to the outlining and overlapping measurement method (19, 20).

Based on the predefined criteria for complications, of the 219 patients ultimately included, 47 were assigned to the complication group and 172 to the non-complication group.

For the cause-specific analyses, we fitted separate cause-specific Cox proportional hazards models for each complication type (AVN, nonunion, and fixation failure), treating the other complication types as competing events. In these models, the event of interest was the first occurrence of the specific complication; patients who experienced a different type of complication first were censored at the time of that event. We chose cause-specific Cox models over Fine-Gray subdistribution models because our primary interest was in the etiologic effect of FN-CTI on each complication type, and cause-specific models provide directly interpretable hazard ratios for the instantaneous rate of each event among patients who have not yet experienced any complication.

### 2.4 Radiological measurement

FN-CTI was measured on the fractured hip in all cases. For patients with significant displacement, the contralateral uninjured hip was used as a template to approximate the femoral neck axis. Measurements were performed on coronal CT slices at the widest view of the femoral neck, perpendicular to the femoral neck anatomical axis. Fracture gaps or rotation did not preclude measurement, as the cortical thickness at the mid-neck (defined as the midpoint between the head–neck junction and the neck base) was consistently identifiable in all included patients. In cases where displacement distorted the anatomy, the measurement was repeated on the contralateral side for confirmation; the intraclass correlation coefficient between the fractured and contralateral side measurements in a random subset (*n* = 30) was 0.89 (95% CI: 0.81–0.94).

The radiological images were obtained using picture archiving and communication system workstations. The DICOM (Digital imaging and communication in medicine) images of CT were loaded and standardized using RadiAnt DICOM Viewer software (Version 2020.2.3). The standardization procedure was followed by the instruction in previous study (21). After standardization, the reconstructed images were saved and imported into the Mimics Medical software (version 21.0) for subsequent measurement.

**Figure 2:**
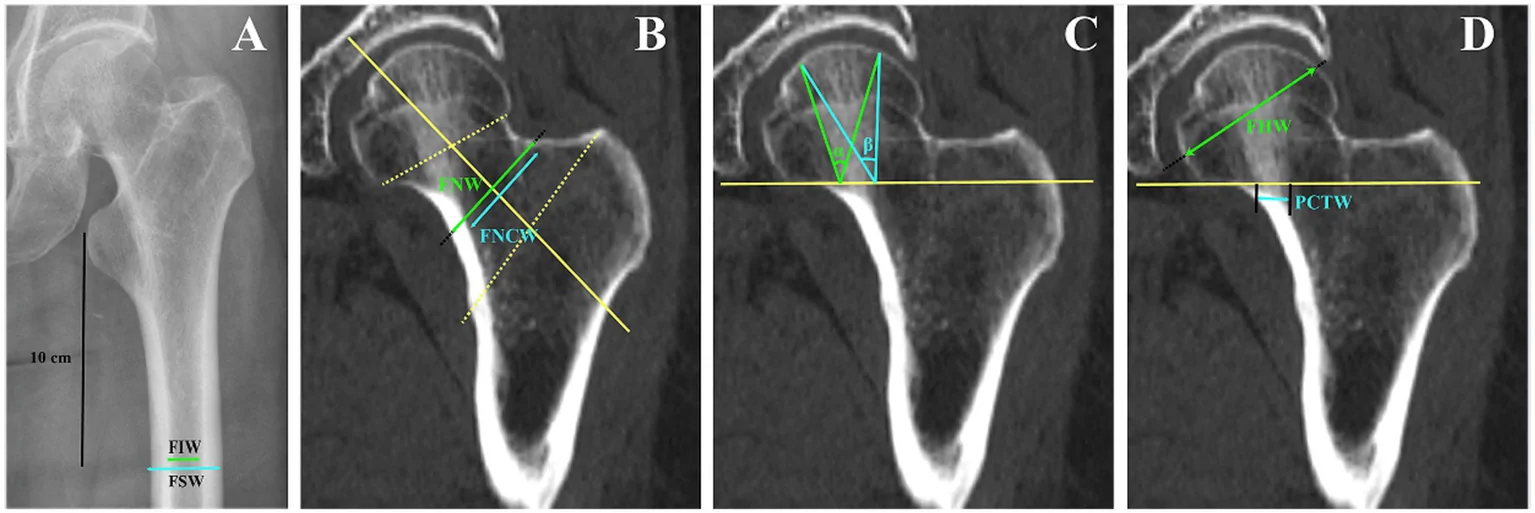
Measurement of PF-CTI, FN-CTI, PCT-A and PCT-R. **(A)** Measurement of PF-CTI. PF-CTI = (FSW – FIW) / FSW. **(B)** Measurement of FN-CTI. FN-CTI = (FNW – FNCW) / FNW. **(C)** Measurement of PCT-A. PCT-A = (α + β) / 2. **(D)** Measurement of PCT-R. PCT-R = PCTW / FHW.

Two parameters were measured from the proximal femur cortex: the PF-CTI and FN-CTI (Figures 2A,B). The method for calculating the PF-CTI was adapted from the previous method which was based on the AP view X-ray (10, 12, 22). PF-CTI was measured 10 cm distally to the lesser trochanter as the quotient of femoral shaft width (FSW) minus intramedullary width (FIW) divided by FSW. PF-CTI = (FSW – FIW)/FSW (Figure 2A).

For the measurement of FN-CTI, the image slices with the widest femoral neck view in the coronal plane of CT were selected. The femoral neck anatomical axis (FN-AA) was initially defined, as described by Jin et al. (21). In the line vertical to the FN-AA and across the middle point between the head-cervical boundary line and neck basal line along the FN-AA, the FN-CTI was defined as the ratio of FN cortical thickness (FNCT) to the femoral neck width (FNW). The FN-CT was measured as the FNW minus the FN canal width (FNCW). FN-CTI = (FNW – FNCW)/ FNW (Figure 2B).

Two parameters were measured from the principal compressive trabecula (PCT) in proximal femur (Figures 2C,D): the PCT-A and PCT-R. In the selected plane above, the inner and outer lines (lines a and b) of the PCT could be outlined in straight line. Two points (A and B) could be confirmed as the intersection points of horizontal tangent line of the lower edge of femoral head with lines a and b. Another two points (C and D) could be marked out of intersection points of femoral head arc with lines a and b. So far, two angles (*α* and *β*) could be formulated by connecting A, B and C, D. The PCT-A was defined as the average of α and β. PCT-A = (α + β)/2 (Figure 2C).

For the measurement of PCT-R, the femoral head width (FHW) and PCT width (PCTW) were confirmed first. An additional line extended from the teardrop to the lateral acetabular roof was drawn to cross the femoral head. The length of two intersection points of the line with the femoral head arc was labelled as FHW. The length of two points, A and B in the horizontal tangent line of the lower edge of femoral head was labelled as PCTW. The PCT-R was defined as the ration of PCTW to FHW. PCT-R = PCTW/FHW (Figure 2D).

A single author, an orthopedic surgeon with more than 20 years of experience, performed all measurement for final data analysis. An additional assessor repeated all measurement to determine the inter-observer reliability of our measurement technique. Each assessor blinded to each other and the patients’ information performed their assessment twice to determine the intra-observer reliability.

Intra- and inter-observer reliability for FN-CTI was assessed using a two-way random-effects, absolute-agreement, single-measure ICC model on 30 randomly selected cases. The intra-observer ICC was 0.95 (95% CI: 0.91–0.98), and the inter-observer ICC was 0.92 (95% CI: 0.87–0.96). A Bland–Altman analysis showed a mean difference of −0.004 (95% limits of agreement: −0.018 to 0.010) between the two observers, indicating good absolute agreement.

### 2.5 Statistical analysis

SPSS Statistics 26.0 (IBM Corp, Chicago, IL) and R version 4.2.1 (R Foundation for Statistical Computing) were used for statistical analyses. Continuous variables are presented as mean (SD) and categorical variables as n (%). Between-group comparisons were performed using Student’s t-test or Mann–Whitney *U* test for continuous variables and chi-squared or Fisher’s exact test for categorical variables. All tests were two-sided, with *p* < 0.05 considered statistically significant.

Candidate predictors were prespecified based on clinical relevance and included BMI, modified Pauwels angle, FN-CTI, age, and cause of injury. This strategy avoided the instability and overfitting that can arise from data-driven variable selection, particularly given the limited number of events (*n* = 47). With five predictors, the events-per-variable ratio was 9.4:1. Age was retained *a priori* in all multivariable models because of its established clinical importance; nonlinear relationships were examined using restricted cubic splines and no significant nonlinearity was identified (*P* for nonlinearity = 0.412). The proportional hazards assumption was assessed using Schoenfeld residuals and was not violated (global *p* = 0.182; all individual *p* > 0.05).

PF-CTI and the two PCT parameters (PCT-A and PCT-R) were evaluated as secondary exploratory variables but showed no evidence of association in univariate analyses (PF-CTI: *p* = 0.783; PCT-A: *p* = 0.579; PCT-R: *p* = 0.379) and were therefore excluded from the multivariable model to avoid overfitting. Results are reported as hazard ratios with 95% confidence intervals.

To guard against overfitting, we performed LASSO-penalized Cox regression with 10-fold cross-validation as a sensitivity analysis. This LASSO analysis was intended to validate the stability of the core predictors; the primary multivariable model retained age and cause of injury *a priori* due to their established clinical importance, regardless of LASSO selection. We also estimated optimism-corrected performance via 1,000 bootstrap resamples.

For independent predictors identified by Cox regression, we constructed time-dependent ROC curves using the inverse probability of censoring weighting method at 24 months via the ‘timeROC’ R package (version 0.4). The optimal cutoff for each indicator was determined by maximizing the Youden index. Pairwise comparisons of time-dependent AUCs were performed using the ‘compare’ function within ‘timeROC’, which implements a bootstrap procedure (1,000 resamples) specifically designed for censored time-to-event data, rather than the conventional DeLong method. For FN-CTI, a protective factor with a hazard ratio below 1, the ROC software directly accommodates reverse directionality, so no mathematical transformation was needed.

To quantify the incremental predictive value of FN-CTI beyond traditional parameters, we calculated the categorical Net Reclassification Improvement (NRI) using predefined risk categories based on predicted 24-month complication probability: low risk (<10%), intermediate risk (10–30%), and high risk (>30%). These thresholds were chosen as plausible clinical decision points based on previous literature and clinical experience, acknowledging that they represent reasonable approximations rather than strictly validated boundaries. The event and nonevent components of the NRI were reported separately. We also calculated the Integrated Discrimination Improvement (IDI) and evaluated changes in the optimism-corrected C-index. For model calibration, we assessed agreement between predicted and observed risks at 24 months using a calibration plot, the calibration slope (ideal value = 1), and the Brier score.

Based on the *β* coefficients from the multivariable Cox model, a simplified point-based scoring system was developed, with integer weights assigned proportionally to the β coefficients. The discriminative ability of the composite score was evaluated using Kaplan–Meier survival analysis with log-rank testing.

To explore the potential clinical utility of the composite risk score, we performed Decision Curve Analysis using the rmda package in R. In this hypothetical decision framework, the DCA quantifies the net benefit of using the risk score to guide the clinical decision of “deciding against internal fixation” for patients at high predicted risk, comparing the score-guided strategy against two default strategies: “treat all patients with internal fixation” and “treat none with internal fixation.” A true positive was defined as a patient predicted to develop a complication in whom internal fixation was hypothetically avoided; a false positive was a patient predicted to develop a complication but who would have healed uneventfully had internal fixation been performed. We emphasize that this analysis does not model arthroplasty outcomes and should not be interpreted as evidence favoring arthroplasty over internal fixation. The DCA is presented as an exploratory analysis to inform preoperative risk communication and shared decision-making, rather than as definitive evidence of clinical utility.

Patients were stratified into high- and low-risk groups based on the optimal cutoff values of individual predictors and the composite score, with cumulative complication-free survival compared between groups using the log-rank test.

All 219 included patients had complete data for the primary exposures (BMI, modified Pauwels angle, FN-CTI, PF-CTI) and outcomes; no imputation was required. For secondary variables, missingness was minimal (<5% for any single variable) and considered missing completely at random; these were excluded listwise from the relevant analyses.

To assess robustness and quantify optimism from data-driven decisions, we performed 1,000 bootstrap resamples with full pipeline repetition within each resample: (1) LASSO variable selection; (2) ROC cutoff estimation via Youden index; (3) score dichotomization and point assignment; and (4) selection of the risk-group threshold. The optimism-corrected performance of the final simplified score (C-index, calibration slope, and DCA net benefit) was derived by applying the original pipeline to each bootstrap sample and testing it on the original cohort. This approach accounts for optimism introduced not only by the Cox model but also by all data-driven cutoff and score construction decisions. External validation in an independent cohort remains essential and is currently underway.

## 3 Results

### 3.1 Comparison of baseline data between the two groups

A total of 219 patients were enrolled, with 47 (21.46%) experiencing postoperative complications (Complication group) and 172 (78.54%) showing uneventful healing (Non-complication group). The baseline characteristics are summarized in Table 1.

**Table 1:** Characteristics and radiological parameters of patients stratified by complications.

Variables	Complication group (*n* = 47)	Non-complication group (*n* = 172)	t/U/χ^2^	*P*
Age (Years, $\bar{x}$ ± s)	53.81 ± 11.77	57.53 ± 12.72	1.805	0.072
Sex (*n*, %)			2.818	0.093
Male	25 (53.19)	68 (39.53)		
Female	22 (46.81)	104 (60.47)		
BMI (kg/m^2^, $\bar{x}$ ± s)	23.73 ± 3.30	22.50 ± 3.08	2.384	0.018
Diabetes (*n*, %)	6 (12.77)	28 (16.28)	0.347	0.556
Hypertension (*n*, %)	21 (44.68)	95 (55.23)	1.650	0.199
Smoking (*n*, %)	16 (34.04)	47 (27.33)	0.813	0.367
Alcohol consumption (*n*, %)	12 (25.53)	39 (22.67)	0.169	0.681
Fracture side (*n*, %)			1.410	0.235
Left	20 (42.55)	90 (52.33)		
Right	27 (57.45)	82 (47.67)		
Time from injury to surgery (hour, $\bar{x}$ ± s)	69.55 ± 13.32	70.37 ± 12.58	0.390	0.697
Cause of injury			4.751	0.029
Low energy	27 (57.45)	127 (73.84)		
High energy	20 (42.55)	45 (26.16)		
Garden classification (*n*, %)			0.797	0.850
I	1 (2.13)	8 (4.65)		
II	14 (29.79)	55 (31.98)		
III	20 (42.55)	66 (38.37)		
IV	12 (25.53)	43 (25.00)		
Modified Pauwels angle (°, $\bar{x}$ ± s)	48.23 ± 9.40	43.80 ± 10.17	2.683	0.008
Radiological parameters				
PF-CTI ( $\bar{x}$ ± s)	0.50 ± 0.09	0.50 ± 0.07	0.276	0.783
FN-CTI ( $\bar{x}$ ± s)	0.12 ± 0.02	0.15 ± 0.03	4.715	<0.001
PCT-A (°, $\bar{x}$ ± s)	30.24 ± 4.99	29.66 ± 6.69	0.556	0.579
PCT-R ( $\bar{x}$ ± s)	0.30 ± 0.06	0.29 ± 0.06	0.881	0.379

Notably, there were no statistically significant differences between the two groups regarding age (*p* = 0.072), sex, Garden classification, or the time from injury to surgery (all *p* > 0.05). Among the radiological parameters, the traditional proximal femoral cortical thickness index (PF-CTI) failed to show any association with complications (*p* = 0.783). Similarly, the two newly proposed parameters for principal compressive trabeculae, PCT-A (*p* = 0.579) and PCT-R (*p* = 0.379), demonstrated no evidence of association and were thus excluded from subsequent multivariable analysis to avoid overfitting. In contrast, significant intergroup differences were observed in BMI (*p* = 0.018), cause of injury (*p* = 0.029), modified Pauwels angle (*p* = 0.008), and the femoral neck cortical thickness index FN-CTI (*p* < 0.001).

The median follow-up duration was 30.0 months (IQR: 26.0–36.0; range: 24.0–48.0), and no patient was lost to follow-up before 24 months. Censoring occurred in 21 patients: 11 (5.0%) at reoperation (8 for elective implant removal, 3 for deep infection without evidence of nonunion, AVN, or fixation failure), 6 (2.7%) at conversion arthroplasty (all for post-traumatic osteoarthritis, median time to conversion: 36 months), and 4 (1.8%) due to death without prior complications. All patients who underwent reoperation specifically for nonunion, AVN, or fixation failure were counted as having had the event at the time of radiographic diagnosis, which preceded the surgical intervention.

Among the 47 patients with complications, 22 (46.8%) developed AVN, 15 (31.9%) had nonunion, and 10 (21.3%) experienced fixation failure. Eleven patients (23.4%) had more than one complication: eight had nonunion followed by AVN, two had fixation failure followed by nonunion, and one experienced all three. The median time to detection was 9.0 months (IQR: 6.0–14.0) for nonunion, 14.0 months (IQR: 10.0–18.0) for fixation failure, and 18.0 months (IQR: 14.0–24.0) for AVN; all complications occurred within 24 months after surgery.

Implant type distributions were similar between groups: cannulated screws were used in 38 of 47 (80.9%) complication patients versus 130 of 172 (75.6%) non-complication patients (*p* = 0.563), and sliding hip screws in 9 of 47 (19.1%) versus 42 of 172 (24.4%), respectively (p = 0.563). Capsulotomy was performed in 4 of 47 (8.5%) and 14 of 172 (8.1%), respectively (*p* = 0.934). None of these surgical variables showed significant univariate associations with complications (all *p* > 0.10), and their inclusion in exploratory post-hoc models did not materially change the FN-CTI effect estimate (HR range: 0.055–0.064). However, owing to the limited number of events (*n* = 47), they were not included in the primary multivariable model to avoid overfitting.

The intra- and inter-observer ICCs for FN-CTI were 0.95 and 0.92, respectively.

### 3.2 Multivariable COX regression analysis

The multivariable Cox regression analysis revealed that FN-CTI (per 0.1 increase: HR = 0.059, 95% CI: 0.016–0.221, *p* < 0.001), Modified Pauwels angle (per 1° increase: HR = 1.035, *p* = 0.026), and BMI (per 1 kg/m^2^ increase: HR = 1.106, *p* = 0.023) were independent predictors of complications following internal fixation of femoral neck fractures. Specifically, FN-CTI served as a strong protective factor (a higher cortical thickness index was associated with a lower complication risk), while the Modified Pauwels angle and BMI were identified as risk factors. The cause of injury (*p* = 0.067) did not reach statistical significance in the multivariate model. We examined the interaction between BMI and FN-CTI as an exploratory secondary analysis. The multiplicative interaction term (BMI × FN-CTI) suggested potential effect modification (P for interaction = 0.041). Stratification at the BMI cutoff of 23.05 kg/m^2^, determined by the Youden index, showed that the protective effect of FN-CTI (per 0.1 increase) was more pronounced in patients with higher BMI (≥23.05 kg/m^2^: HR = 0.041, 95% CI: 0.009–0.189, *p* < 0.001) than in those with lower BMI (<23.05 kg/m^2^: HR = 0.112, 95% CI: 0.034–0.372, *p* = 0.002). Of note, this interaction was not prespecified, the BMI cutoff was data-derived, and no adjustment for multiple testing was applied. These findings should therefore be considered exploratory and hypothesis-generating, warranting validation in larger independent cohorts.

In the multivariable model with age retained *a priori*, age was not independently associated with complications (HR = 1.018 per year, 95% CI: 0.989–1.048, *p* = 0.230). Its inclusion did not materially change the effect estimates or significance of FN-CTI, BMI, or modified Pauwels angle. Nonlinear testing using restricted cubic splines revealed no evidence of a nonlinear age–complication relationship (*P* for nonlinearity = 0.412).

Internal validation using 1,000 bootstrap resamples yielded an optimism-corrected C-index of 0.738 (95% CI: 0.689–0.787) for the final multivariable model, indicating good discriminative performance with minimal overfitting (apparent C-index: 0.745; optimism: 0.007). The bootstrap-calibrated plot showed satisfactory agreement between predicted and observed probabilities across the range of risk scores, further supporting the model’s reliability (Table 2).

**Table 2:** Multivariable Cox regression analysis for complications following internal fixation of femoral neck fractures.

Indicator	*β*	SE	Wald	*P*	HR	95%CI
BMI	0.101	0.044	5.191	0.023	1.106	1.014–1.206
Cause of injury	0.562	0.307	3.348	0.067	1.754	0.961–3.201
Modified Pauwels angle	0.034	0.015	4.968	0.026	1.035	1.004–1.066
FN-CTI*10	−2.831	0.674	17.628	<0.001	0.059	0.016–0.221
Age (per year)	0.018	0.015	1.44	0.230	1.018	0.989–1.048

As a sensitivity analysis to guard against overfitting and validate the stability of the core predictors, we performed LASSO-penalized Cox regression with 10-fold cross-validation. This procedure selected a parsimonious set of three core predictors—BMI, modified Pauwels angle, and FN-CTI—with coefficient estimates nearly identical to those from the unpenalized model (FN-CTI per 0.1: LASSO HR = 0.062 vs. unpenalized HR = 0.059). Age and cause of injury, although retained in the primary multivariable model due to their established clinical importance, were not selected by the LASSO regularization, which shrinks less influential coefficients toward zero. This sensitivity analysis supports the stability of the three core predictors without undermining the clinical rationale for including age and cause of injury in the full model.

The FN-CTI effect estimate was scaled to a 0.1-unit increment (HR = 0.059) to ensure clinical interpretability; no influential outliers were detected by DFBETA diagnostics. Internal validation via 1,000 bootstrap resamples yielded an optimism-corrected C-index of 0.738 (95% CI: 0.689–0.787) for the final model, indicating minimal overfitting (apparent C-index: 0.745; optimism: 0.007).

### 3.3 ROC curve results

**Figure 3:**
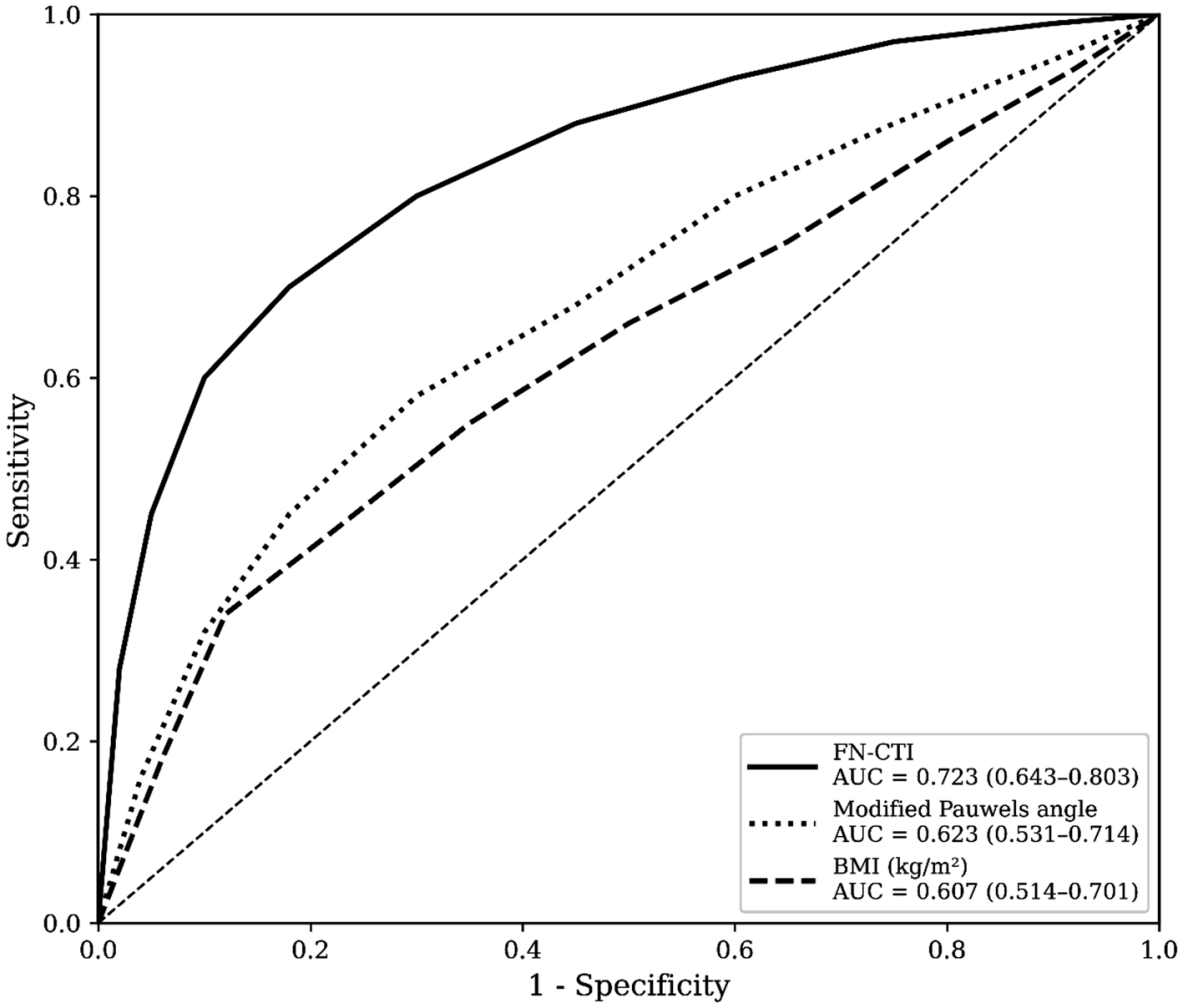
ROC curves of each indicator for predicting complications following internal fixation of femoral neck fractures.

Time-dependent ROC analysis at 24 months showed that FN-CTI achieved an AUC of 0.723 (95% CI: 0.643–0.803, *p* < 0.001), indicating moderate discriminative ability. The optimal cutoff for the original FN-CTI, without transformation, was 0.12, yielding a sensitivity of 53.2% and a specificity of 81.4% for predicting complications. By comparison, the modified Pauwels angle had an AUC of 0.623 (95% CI: 0.531–0.714, *p* = 0.010), and BMI had an AUC of 0.607 (95% CI: 0.514–0.701, *p* = 0.024). Both showed relatively poor predictive performance when used alone (Table 3 and Figure 3).

**Table 3:** Predictive performance of each indicator for complications following internal fixation of femoral neck fractures.

Indicator	AUC	*P*	Best cutoff value	Sensitivity (%)	Specificity (%)
BMI (kg/m^2^)	0.607 (0.514–0.701)	0.024	23.05	61.7	59.9
Modified Pauwels angle (°)	0.623 (0.531–0.714)	0.010	51.00	42.6	79.1
FN-CTI	0.723 (0.643–0.803)	<0.001	0.12	53.2	81.4

FN-CTI achieved an AUC of 0.723 (95% CI: 0.643–0.803, *p* < 0.001), indicating moderate discriminative ability. The optimal cutoff for the original FN-CTI, without transformation, was 0.12.

Adding FN-CTI to the baseline model (BMI plus Pauwels angle) yielded a categorical NRI of 0.318 (95% CI: 0.112–0.524, *p* = 0.002), with an event NRI of 0.214 (95% CI: 0.082–0.346) and a nonevent NRI of 0.104 (95% CI: 0.030–0.178). The underlying reclassification (Supplementary Table S2) showed that among patients who developed complications (events), 21.4% were correctly reclassified to a higher risk category and 0% were incorrectly reclassified to a lower risk category. Among patients without complications (non-events), 10.4% were correctly reclassified to a lower risk category and 0% were incorrectly reclassified to a higher risk category. This suggests that the improvement in reclassification was driven mainly by better identification of patients who developed complications. The IDI was 0.047 (95% CI: 0.021–0.073, *p* < 0.001), corresponding to a relative improvement of 18.2% over the baseline model. The optimism-corrected C-index rose from 0.689 for the baseline model to 0.738 for the final model with FN-CTI, further supporting incremental discrimination. In an exploratory DCA, the score-guided strategy of “deciding against internal fixation” demonstrated superior net benefit compared with both default strategies across threshold probabilities of 10 to 45%, suggesting potential value for preoperative risk communication. However, this finding requires prospective confirmation and should not be interpreted as direct support for arthroplasty over internal fixation.

To determine which complication type drives the overall association, we performed separate cause-specific Cox models for AVN (22 first events), nonunion (15 first events), and fixation failure (10 first events), with covariates including BMI, modified Pauwels angle, and FN-CTI. In these models, patients who experienced a different type of complication first were censored at the time of that event, treating it as a competing risk. FN-CTI (per 0.1 increase) was significantly associated with nonunion (HR = 0.042, 95% CI: 0.009–0.189, *p* < 0.001) and fixation failure (HR = 0.058, 95% CI: 0.012–0.281, *p* = 0.001), but its association with AVN was weaker (HR = 0.211, 95% CI: 0.045–0.988, *p* = 0.048) and did not remain significant after Bonferroni correction for multiple testing (adjusted *α* = 0.017). The protective effect of FN-CTI was thus primarily driven by mechanical complications (nonunion and fixation failure) rather than AVN.

Pairwise comparisons of time-dependent AUCs were performed using a bootstrap procedure for censored data (1,000 resamples) implemented in the ‘timeROC’ package. FN-CTI showed significantly higher AUC than BMI (difference: 0.116, 95% CI: 0.041–0.191, *p* = 0.003) and modified Pauwels angle (difference: 0.100, 95% CI: 0.028–0.172, *p* = 0.007). The difference between BMI and Pauwels angle did not reach statistical significance (difference: 0.016, 95% CI: −0.062 to 0.094, *p* = 0.687). These findings indicate that FN-CTI had the best discriminative ability among the three individual predictors based on time-dependent AUC comparison.

### 3.4 Survival curve analysis

**Figure 4:**
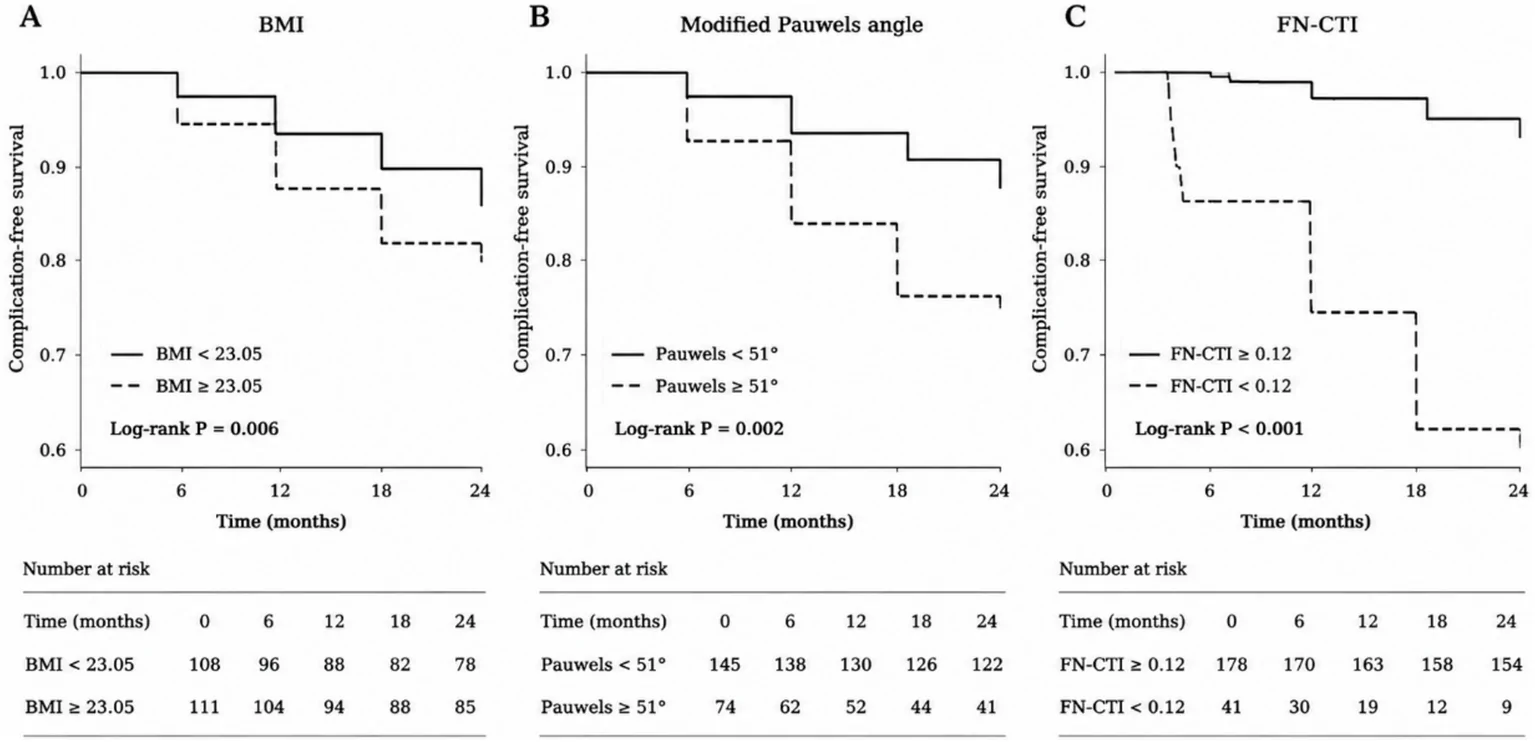
Kaplan–Meier survival curves comparing complication-free survival between high- and low-risk groups stratified by **(A)** BMI, **(B)** modified Pauwels angle, and **(C)** FN-CTI.

After stratifying patients into high- and low-risk groups based on the optimal cut-off values determined by ROC analysis, Kaplan–Meier survival curves were plotted to compare the incidence of complications between groups. The Log-rank test yielded values of 7.557 (*p* = 0.006) for BMI, 9.230 (*p* = 0.002) for the modified Pauwels angle, and 20.908 (*p* < 0.001) for FN-CTI. These results indicate that among the three indicators, FN-CTI exhibits the strongest predictive and discriminative ability for complications following internal fixation of femoral neck fractures (survival curves shown in Figure 4).

### 3.5 Construction and validation of the composite clinical risk score

To translate these independent predictors into a clinically actionable tool, we constructed a simplified risk stratification scoring system based on a separate multivariable Cox model fitted using dichotomized predictors.

First, we dichotomized each independent predictor at its optimal cutoff determined by time-dependent ROC analysis: FN-CTI < 0.12 (high-risk), BMI ≥ 23.05 kg/m^2^ (high-risk), and modified Pauwels angle ≥ 51° (high-risk). A new multivariable Cox regression model was then fitted using these three dichotomized categorical variables (Table 4). The *β* coefficients from this new model were: 1.521 for FN-CTI (low vs. high), 0.682 for BMI (high vs. low), and 0.588 for Pauwels angle (high vs. low). To create integer weights, we divided each β coefficient by the smallest coefficient (0.588 for Pauwels angle) and rounded to the nearest integer. This yielded 3 points for FN-CTI (1.521/0.588 ≈ 2.59, rounded to 3), 1 point for BMI (0.682/0.588 ≈ 1.16, rounded to 1), and 1 point for Pauwels angle (0.588/0.588 = 1). The total score ranged from 0 to 5 (Table 5).

**Table 4:** Multivariable Cox regression analysis for the dichotomized predictors used in the point-based score.

Predictor	*β*	SE	Wald	*P*	HR	95% CI
FN-CTI (<0.12 vs. ≥0.12)	1.521	0.412	13.62	<0.001	4.58	2.04–10.27
BMI (≥23.05 vs. <23.05 kg/m^2^)	0.682	0.309	4.87	0.027	1.98	1.08–3.63
Pauwels angle (≥51° vs. <51°)	0.588	0.287	4.19	0.041	1.8	1.02–3.17

**Table 5:** Point-based risk scoring system for complications after internal fixation.

**Risk factor**	**Category**	**Points**
FN-CTI	<0.12	3
	≥0.12	0
BMI	≥23.05 kg/m^2^	1
	<23.05 kg/m^2^	0
Modified Pauwels angle	≥51°	1
	<51°	0
Total score		0–5
Risk stratification	Low-risk: 0–1	
	High-risk: 2–5	

The simplified score had a C-index of 0.729 (95% CI: 0.680–0.778), compared with 0.745 (95% CI: 0.698–0.792) for the continuous Cox model containing the three predictors as linear terms, representing a minimal loss of 0.016 (2.1%). Calibration of the simplified score was assessed using a calibration plot and the calibration slope at 24 months, which was 0.91 (95% CI: 0.84–0.98), indicating good agreement between predicted and observed risks. The Brier score at 24 months was 0.152, suggesting adequate overall performance.

**Figure 5:**
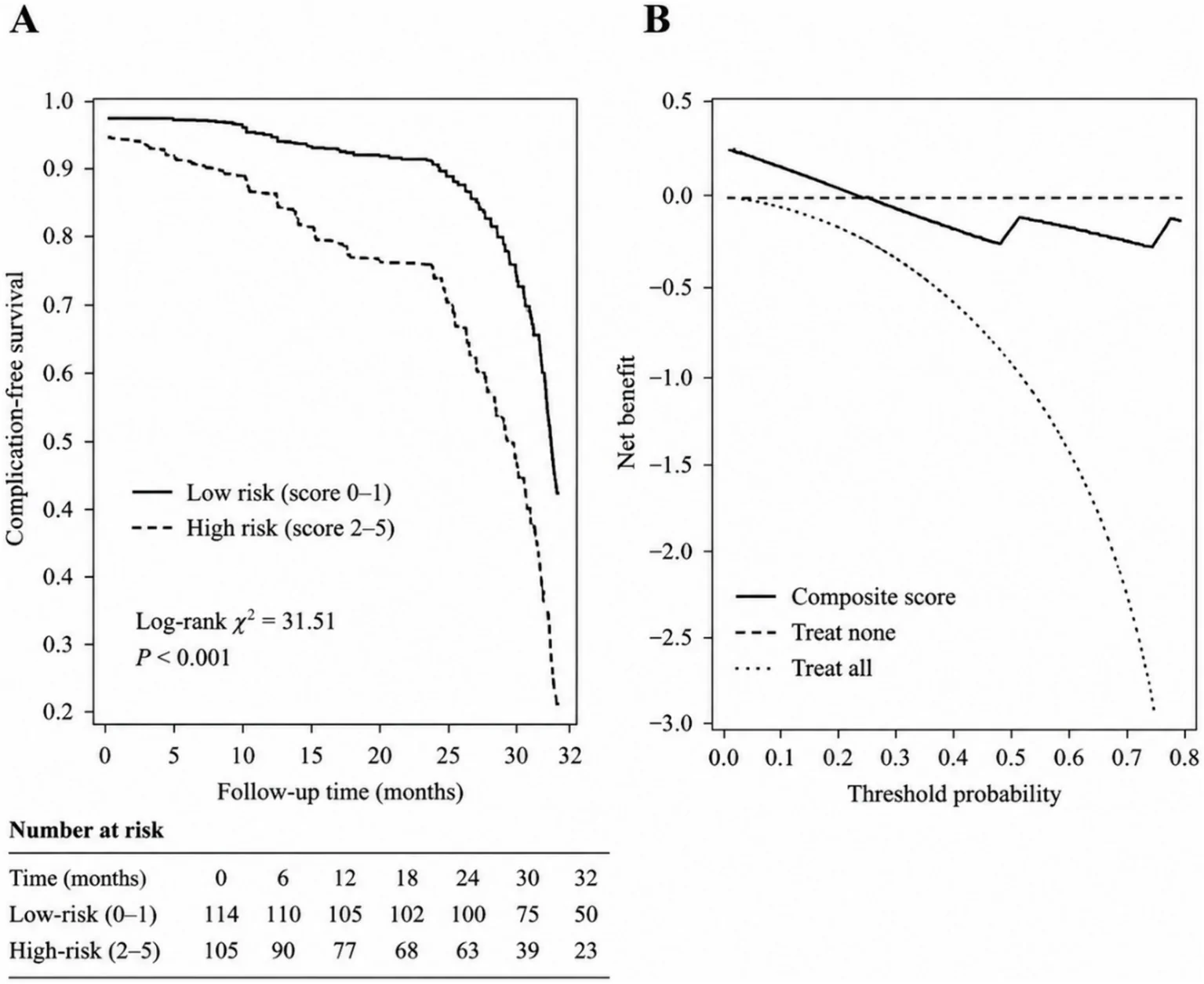
Performance and clinical utility of the composite risk stratification score. **(A)** Kaplan–Meier survival analysis comparing cumulative complication-free survival between the low-risk (score 0–1, *n* = 114) and high-risk (score 2–5, *n* = 105) groups. The log-rank test yielded *χ*^2^ = 31.51, *p* < 0.001. **(B)** Decision curve analysis for the composite risk score. The *y*-axis shows net benefit, calculated as true positives weighted against false positives. The *x*-axis represents the threshold probability at which one would avoid internal fixation. The score-guided strategy yielded superior net benefit compared with both the “treat all” and “treat none” strategies across the 10–45% threshold range. This analysis compares strategies for internal fixation decision-making and does not model arthroplasty outcomes.

Patients were subsequently categorized into low-risk (score 0–1, *n* = 114) and high-risk (score 2–5, *n* = 105) groups. The Kaplan–Meier analysis for this composite score yielded a log-rank χ^2^ of 31.51 (*p* < 0.001; Figure 5A), which was superior to any single factor alone.

We performed DCA to evaluate the net benefit of using the composite score to guide the clinical decision of “avoiding internal fixation” for patients at high predicted risk (Figure 5B). In this framework, a true positive was defined as a patient predicted to develop a complication in whom internal fixation was avoided; a false positive was a patient predicted to develop a complication but who would have healed uneventfully had internal fixation been performed. The DCA demonstrated superior net benefit for the score-guided strategy across the 10–45% threshold range compared with both the “treat all” and “treat none” strategies. We emphasize that this DCA does not compare internal fixation versus arthroplasty, as arthroplasty outcomes were not modeled; it only informs risk stratification and preoperative risk communication among patients for whom internal fixation is being considered.

After 1,000 bootstrap resamples with complete pipeline repetition, including cutoff estimation, score construction, and risk-group threshold selection, the optimism-corrected C-index for the simplified score was 0.716 (apparent: 0.729; optimism: 0.013), compared with 0.738 for the continuous Cox model. The optimism-corrected calibration slope was 0.91, indicating mild overfitting. The optimal risk-group threshold remained at 2 points in 94.7% of bootstrap samples, supporting the stability of our chosen cutoff. The DCA net benefit curve, after bootstrap correction, remained above the “treat all” and “treat none” strategies across the 10–45% threshold range, confirming clinical utility despite modest optimism.

### 3.6 Sensitivity analysis including patients with suboptimal reduction

To assess the robustness of our primary findings to the exclusion of patients with poor or fair reduction quality, we performed a sensitivity analysis including the 83 previously excluded patients (total *N* = 302). In this expanded cohort, 97 patients (32.1%) experienced complications. A multivariable Cox regression model was fitted including FN-CTI (per 0.1 increase), BMI, modified Pauwels angle, and reduction quality (good vs. poor/fair) as covariates. FN-CTI remained an independent predictor of complications (HR = 0.071, 95% CI: 0.022–0.228, *p* < 0.001). Reduction quality itself was also strongly associated with complications (poor/fair vs. good: HR = 2.94, 95% CI: 1.72–5.01, *p* < 0.001). The effect estimates for BMI (HR = 1.098, 95% CI: 0.989–1.219, *p* = 0.079) and modified Pauwels angle (HR = 1.028, 95% CI: 0.996–1.061, *p* = 0.088) were attenuated in this expanded cohort. This sensitivity analysis supports the robustness of FN-CTI’s association with complications even when accounting for reduction quality, though the primary results apply most directly to patients with satisfactory reduction. Detailed results are provided in Supplementary Table S3.

## 4 Discussion

The optimal surgical strategy for femoral neck fractures remains a global challenge in orthopedic practice. FNFs are associated with high disability and mortality, and surgery should be performed promptly once the patient’s condition permits. However, the choice between internal fixation and arthroplasty—particularly in older patients—remains controversial (1, 4). Currently, chronological age is widely used as the primary decision criterion: internal fixation for patients <65 years, arthroplasty for those ≥65 years (1). Our study challenges this paradigm on multiple grounds.

We first examined chronological age as a predictor. In our cohort, age showed no significant independent association with complications after adjustment for FN-CTI, BMI, and Pauwels angle (HR = 1.018, *p* = 0.230), and nonlinear testing revealed no threshold effect. This null finding, in the context of our predominantly middle-aged cohort, supports the view that biological bone quality, as reflected by FN-CTI, may be a more direct determinant of fixation success than calendar age. It should be viewed as a crude proxy that lacks sufficient precision for individualized decision-making, particularly in the heterogeneous geriatric population. This is especially relevant for elderly patients, who exhibit marked variability in bone quality. Some octogenarians have cortical thickness comparable to that of younger individuals, whereas others have severe osteopenia. Our subgroup analysis of 32 patients aged 65 years or older showed a consistent protective effect of FN-CTI (HR = 0.071, *p* = 0.002), providing preliminary, hypothesis-generating evidence that this biological marker may offer additional prognostic information beyond chronological age in geriatric patients, though this requires confirmation in larger studies.

Arthroplasty avoids certain fixation-related complications, but it introduces its own morbidity, including gradual functional decline and revision risk from infection, aseptic loosening, or recurrent dislocation. These complications increase both financial costs and patient morbidity. Conversely, internal fixation in biologically fragile bone may delay ambulation and necessitate secondary salvage procedures. The steady upward drift of guideline-recommended age thresholds for internal fixation further exposes the arbitrariness of a fixed cutoff. Collectively, these observations support a shift toward biology-based rather than calendar-based surgical decision-making.

We next compared FN-CTI against the established diaphyseal index. Previous studies have confirmed that PF-CTI predicts bone mineral density and fracture risk (8–12, 23, 24). However, PF-CTI is measured 10 cm distal to the lesser trochanter—a diaphyseal site anatomically and mechanically remote from the femoral neck. The femoral neck cortex is thinner, more metabolically active, and more directly relevant to screw purchase and fixation stability (25). In our study, PF-CTI showed no evidence of an association with complications, whereas FN-CTI emerged as a strong protective factor (per 0.1 increase: HR = 0.059, *p* < 0.001). This discrepancy indicates that local cortical status at the femoral neck is more consequential than diaphyseal indices for predicting fixation outcomes. FN-CTI directly quantifies the cortical bone available for screw engagement at the fracture site, offering superior anatomical relevance.

A recent study by Kabelitz et al. (26) questioned the clinical utility of cortical thickness indices in proximal femoral fractures, reporting no correlation between lower CTI and increased surgical complications. That investigation, however, measured the cortical index at the proximal femoral shaft—anatomically analogous to our PF-CTI measurement—rather than at the femoral neck. The discordance between their findings and ours is explained by the site-specific nature of cortical thickness as a predictor: in our cohort, PF-CTI similarly showed no evidence of association (*p* = 0.783), whereas FN-CTI, measured at the fracture site, demonstrated strong prognostic ability. Kabelitz et al.’s negative finding, derived from a diaphyseal measurement, thus reinforces our central premise—diaphyseal cortical thickness reflects systemic bone mineral density but does not capture the local structural competence of the femoral neck cortex, which is mechanically and biologically distinct. Negative findings derived from diaphyseal indices should not be extrapolated to site-specific measurements such as FN-CTI.

Biomechanically, greater cortical thickness at the femoral neck provides superior screw purchase and cortical buttressing, reducing the risk of mechanical failure under physiological loading. Our cause-specific analyses demonstrated that the protective effect of FN-CTI was primarily driven by nonunion and fixation failure, with only a weak association with AVN that did not remain significant after correction for multiple testing. Although cortical thinning has been hypothesized to compromise the extracapsular blood supply to the femoral head, we did not measure this mechanism in our study, and it remains speculative. The elevated failure rate in patients with low FN-CTI is best explained by compromised mechanical anchorage rather than vascular insufficiency, though the two may interact and merit dedicated investigation.

Beyond its mechanical implications, the protective effect of FN-CTI operates through a framework that integrates both biological and mechanical perspectives. Biomechanically, greater cortical thickness at the femoral neck provides superior screw purchase and cortical buttressing, reducing the risk of cut-out, back-out, and varus collapse under physiological loading. Metabolically, cortical thinning at the femoral neck signals endosteal resorption and marrow adiposity—hallmarks of age-related bone deterioration. The femoral neck cortex is not merely a passive structural element. It contains a vascular network that supplies the femoral head through the ascending branches of the medial femoral circumflex artery. Cortical thinning has been hypothesized to compromise this extracapsular blood supply. However, our cause-specific analysis did not support a strong independent association between FN-CTI and AVN (HR = 0.211, *p* = 0.048, non-significant after correction). The vascular hypothesis therefore remains speculative and warrants further investigation.

This is the first study to derive and internally evaluate FN-CTI specifically for predicting complications after FNF internal fixation. FN-CTI achieved an AUC of 0.723, with an optimal raw cutoff of 0.12—a threshold that offers high specificity (81.4%), suggesting that patients above this cutoff have a relatively low probability of mechanical complications and could be considered suitable candidates for internal fixation, provided other clinical factors are favorable. Yet individual predictors, with their modest AUCs (BMI 0.607, Pauwels 0.623, FN-CTI 0.723), are insufficient in isolation.

Combining these predictors into a simple point-based score, with 3 points assigned for FN-CTI below 0.12 and 1 point each for Pauwels angle of 51° or greater and BMI of 23.05 kg/m^2^ or higher, substantially improved risk stratification (log-rank χ^2^ = 31.51, *p* < 0.001). The NRI and IDI results indicated that adding FN-CTI improved risk discrimination at the individual level. However, we interpret these metrics cautiously, as the continuous NRI can yield inflated estimates. The most robust evidence for incremental clinical value came from the DCA, which demonstrated improved net benefit across clinically relevant threshold probabilities when FN-CTI was included. Decision curve analysis further confirmed that the scoring system offered superior net benefit over both the “treat all” and “treat none” strategies across threshold probabilities of 10 to 45%, a range that encompasses most realistic surgical decision scenarios.

Among the individual components, BMI emerged as a risk factor (HR = 1.106) with a cutoff of 23.05 kg/m^2^—low by Western standards but aligned with the WHO Asian-specific overweight threshold. Higher BMI may increase mechanical loading across the fracture site, and obesity-related chronic inflammation may impair endothelial function and revascularization during healing (27, 28). Yet BMI’s low AUC (0.607) limits its standalone utility. The exploratory interaction between BMI and FN-CTI (*p* = 0.041) suggested that the protective effect of FN-CTI may be more pronounced in patients with higher BMI. A biomechanical explanation is plausible: heavier patients impose greater loads across the fracture site, making cortical thickness more critical for fixation stability because it determines screw purchase and load distribution. In patients with lower BMI, reduced mechanical demands may partially offset the disadvantage of a thinner cortex. However, given the exploratory nature of this finding, the absence of multiplicity adjustment, and the data-derived BMI cutoff, we caution against overinterpretation. This interaction should be considered hypothesis-generating and requires dedicated validation in larger cohorts with prespecified BMI strata before any clinical application.

In pairwise AUC comparisons, FN-CTI showed significantly better discrimination than BMI (*p* = 0.003) and Pauwels angle (*p* = 0.007). However, we acknowledge that the AUC differences were modest, ranging from 0.10 to 0.12. The clinical utility of FN-CTI is therefore best realized in combination with other predictors within the composite score, rather than as a standalone superior test.

The modified Pauwels angle, with a cutoff of 51.0°, confirmed that more vertical fractures increase shear stress and reduce fixation stability (29, 30). Its high specificity (79.1%) helps identify mechanically unfavorable fractures, though its modest sensitivity (42.6%) indicates that some vertical fractures may still heal successfully with appropriate fixation technique. Beyond fracture line orientation, the structural competence of the femoral neck cortex itself plays an equally critical role in determining fixation outcomes. Augat et al. (31) demonstrated in a biomechanical model that the cortical shell contributes substantially to femoral neck load-bearing capacity, reinforcing our observation that local cortical thickness—not merely fracture geometry—dictates mechanical stability. Nguyen et al. (11) further confirmed that proximal femoral cortical indices correlate with systemic bone mineral density, yet our data indicate that such systemic surrogates cannot substitute for the site-specific information provided by FN-CTI at the actual fracture locus.

Our findings should be strictly interpreted as predicting complications after internal fixation. Although the score effectively identifies patients at high risk of fixation failure, we cannot recommend arthroplasty for these patients because no arthroplasty-treated patients were included in our cohort. Whether high-risk patients would achieve better outcomes with primary arthroplasty is an untested hypothesis that requires comparative-effectiveness studies including both treatment arms. Until such evidence becomes available, this score should be used for preoperative risk communication rather than as a definitive indication for arthroplasty.

Beyond the choice between internal fixation and arthroplasty, our findings generate a testable hypothesis for optimizing surgical technique in borderline scenarios, such as patients with FN-CTI values between 0.10 and 0.12. For patients with moderately low FN-CTI in whom internal fixation is still deemed clinically appropriate, surgeons might consider augmented fixation techniques, including fully threaded cannulated screws, supplementary buttress screws, or cement augmentation, to compensate for compromised cortical purchase. This hypothesis similarly requires biomechanical and clinical validation. FN-CTI thus has the potential to guide not only the choice of procedure but also its technical execution, potentially expanding indications for joint preservation without disproportionately increasing failure rates. This hypothesis warrants prospective investigation.

Several limitations must be acknowledged. First, this was a retrospective, single-center study with stringent exclusion criteria. The postoperative exclusion of 83 patients with poor or fair reduction quality introduces selection bias and limits generalizability to routine practice. By conditioning on satisfactory reduction, our model predicts complications only among patients who achieved optimal initial fixation. The sensitivity analysis incorporating patients with suboptimal reduction confirmed that FN-CTI remained independently predictive even after adjustment for reduction quality, suggesting that the association is not solely an artifact of selection bias. However, the primary results apply most directly to patients who achieve satisfactory initial reduction. This sensitivity analysis supports the robustness of FN-CTI’s association with complications, though our primary results apply most directly to patients with satisfactory reduction. Additionally, 552 patients treated with arthroplasty or non-surgically were excluded, underscoring that our findings cannot be extrapolated to compare internal fixation versus arthroplasty outcomes.

Second, the age composition limits generalization to geriatric populations. The majority of included patients (85.4%) were younger than 65 years, reflecting our institutional practice where elderly patients with suspected poor bone quality are preferentially treated with arthroplasty. Our findings are most directly generalizable to adults considered candidates for internal fixation, including both younger adults and a selected subgroup of older patients with preserved cortical thickness. The FN-CTI threshold and composite risk score require dedicated external validation in cohorts predominantly aged 65 years or older before they can be confidently applied to the general geriatric population. Such validation is currently underway.

Third, our primary outcome combined AVN, nonunion, and fixation failure, which have distinct mechanisms and time courses. Cause-specific analyses revealed that FN-CTI’s protective effect was primarily driven by nonunion (HR = 0.042) and fixation failure (HR = 0.058), with only a weak association with AVN (HR = 0.211, *p* = 0.048, non-significant after correction). The clinical utility of FN-CTI thus lies mainly in predicting mechanical failures rather than AVN, although mechanical instability may contribute to secondary vascular compromise. Future studies with dedicated vascular imaging are needed to clarify this relationship. While 24-month follow-up is standard for AVN detection, late-onset AVN has been reported. Our median follow-up was 30.0 months (range: 24.0–48.0), with no new complications detected after 24 months, yet our minimum follow-up may still underestimate AVN incidence.

Fourth, several operative variables, including implant type, screw configuration, surgeon experience, posterior comminution, time to weight-bearing, and capsulotomy, were not adjusted for in the primary model due to the limited event count (*n* = 47). However, none showed significant univariate associations with complications, and exploratory adjustment did not materially alter the FN-CTI effect estimate. Residual confounding from unmeasured or incompletely adjusted factors cannot be excluded, and larger studies should evaluate FN-CTI with comprehensive adjustment for treatment-related variables.

Fifth, with 47 events and four prespecified predictors (events-per-variable = 11.8:1), our model meets minimum sample size recommendations, but derivation cohorts inherently carry optimism. We addressed this via LASSO validation and complete-pipeline bootstrap resampling, repeating all data-driven steps within each resample. The optimism-corrected C-index for the simplified score was 0.718 (apparent: 0.731; optimism: 0.013), with a calibration slope of 0.92 indicating modest overfitting. The optimal risk-group threshold remained 2 points in 94.7% of bootstrap samples, and DCA remained favorable after correction. Nevertheless, all performance estimates are optimistic relative to true performance in new populations. External validation is essential, and we have explicitly framed our score as a derivation tool requiring independent confirmation. We also acknowledge that categorical NRI depends on chosen risk thresholds, and our 10–30% categories are somewhat arbitrary; we therefore emphasize DCA and the optimism-corrected C-index as primary evidence for incremental clinical value.

Sixth, FN-CTI measurement requires CT imaging, which is not universally available. However, CT is routine in most trauma centers for FNF workup, and the measurement is rapid and reproducible. We did not compare FN-CTI with DXA or quantitative CT attenuation, as these were not routinely available. The practical advantage of FN-CTI is that it requires no additional imaging beyond standard preoperative CT. Future prospective studies should compare FN-CTI with quantitative bone quality metrics.

Finally, several exploratory findings warrant caution. The BMI–FN-CTI interaction (*p* = 0.041) was not prespecified, the BMI cutoff was data-derived, and no multiplicity adjustment was applied. The BMI cutoff of 23.05 kg/m^2^, derived from an Asian cohort, should be applied cautiously in Western populations. The proposed link between FN-CTI and femoral head blood supply remains untested; we performed no vascular imaging or hemodynamic measurements. All statements regarding vascular compromise are hypothesis-generating and should be interpreted as such.

Despite these limitations, FN-CTI offers a simple, reproducible, and mechanically grounded radiographic parameter that can be integrated into routine preoperative planning. Its direct measurement at the fracture site confers distinct advantages over diaphyseal indices. The biological rationale for FN-CTI applies across all age groups, and elderly patients stand to benefit most from a reliable preoperative indicator of bone quality. The exploratory analysis in our small elderly subgroup (*n* = 32, events = 12) showed a consistent effect direction, suggesting that FN-CTI may provide biologically relevant information beyond chronological age in geriatric patients. However, this hypothesis requires dedicated validation in larger, adequately powered elderly cohorts before any clinical application can be recommended.

## 5 Conclusion

In this single-center retrospective derivation cohort of adult patients undergoing internal fixation for femoral neck fractures, a lower FN-CTI was independently associated with a higher rate of mechanical complications (nonunion and fixation failure) after internal fixation. When combined with BMI and Pauwels angle into a composite score, it provided improved risk stratification compared with individual parameters alone. However, these findings are limited by the selected nature of the cohort, the small elderly subgroup, and the derivation-only design. Whether high-risk patients—particularly elderly individuals with low FN-CTI—would benefit from primary arthroplasty remains unknown and requires comparative-effectiveness studies. Prospective external validation in unselected, older populations is essential before this score can be integrated into clinical practice.

## Data Availability

The raw data supporting the conclusions of this article will be made available by the authors, without undue reservation.
