# Supplementary material for: Femoral neck-specific cortical thickness predicts internal fixation complications across adults with implications for elderly surgical decision-making
Source: Front Med (Lausanne). 2026 Aug 27;13:1927915. doi: 10.3389/fmed.2026.1927915 (PMC13558039; doi:10.3389/fmed.2026.1927915)
Supplement: Supplementary file 1 [file Table_1.docx]

**Supplementary Table S1. Exploratory subgroup analysis of patients aged ≥65 years (n=32, events=12)**

| **Variable** | **HR (95% CI)** | **P value** |
| --- | --- | --- |
| FN-CTI (per 0.1 increase) | 0.071 (0.018–0.285) | 0.002 |
| BMI (per 1 kg/m²) | 1.098 (0.957–1.259) | 0.189 |
| Modified Pauwels angle (per 1°) | 1.031 (0.972–1.094) | 0.325 |
