# Supplementary material for: Femoral neck-specific cortical thickness predicts internal fixation complications across adults with implications for elderly surgical decision-making
Source: Front Med (Lausanne). 2026 Aug 27;13:1927915. doi: 10.3389/fmed.2026.1927915 (PMC13558039; doi:10.3389/fmed.2026.1927915)
Supplement: Supplementary file 2 [file Table_2.docx]

**Supplementary Table S2. Reclassification for Addition of FN-CTI to Baseline Model (BMI + Pauwels Angle)**

| **Baseline Model** | **Low Risk (<10%)** | **Intermediate (10–30%)** | **High Risk (>30%)** | **Total** |
| --- | --- | --- | --- | --- |
| **Patients with complications (n=47)** |  |  |  |  |
| **Low Risk (<10%)** | **0** | **0** | **0** | **0** |
| **Intermediate (10–30%)** | **0** | **4** | **10** | **14** |
| **High Risk (>30%)** | **0** | **0** | **33** | **33** |
| **Patients without complications (n=172)** |  |  |  |  |
| **Low Risk (<10%)** | **85** | **5** | **0** | **90** |
| **Intermediate (10–30%)** | **13** | **42** | **2** | **57** |
| **High Risk (>30%)** | **0** | **5** | **20** | **25** |
