# Supplementary material for: Femoral neck-specific cortical thickness predicts internal fixation complications across adults with implications for elderly surgical decision-making
Source: Front Med (Lausanne). 2026 Aug 27;13:1927915. doi: 10.3389/fmed.2026.1927915 (PMC13558039; doi:10.3389/fmed.2026.1927915)
Supplement: Supplementary file 3 [file Table_3.docx]

**Supplementary Table S3. Sensitivity Analysis Including Patients with Poor or Fair Reduction Quality (N=302, Events=97)**

| **Predictor** | **HR** | **95% CI** | **P value** |
| --- | --- | --- | --- |
| **FN-CTI (per 0.1 increase)** | **0.071** | **0.022–0.228** | **<0.001** |
| **BMI (per 1 kg/m²)** | **1.098** | **0.989–1.219** | **0.079** |
| **Modified Pauwels angle (per 1°)** | **1.028** | **0.996–1.061** | **0.088** |
| **Reduction quality (poor/fair vs. good)** | **2.94** | **1.72–5.01** | **<0.001** |
